# Physical Activity and Exercise Practice to Reduce the Sedentary Behavior in Children and Adolescents Overweight and with Obesity

**DOI:** 10.3390/ijerph19105996

**Published:** 2022-05-15

**Authors:** Matteo Vandoni, Vittoria Carnevale Pellino

**Affiliations:** 1 Laboratory of Adapted Motor Activity (LAMA), Department of Public Health, Experimental Medicine and Forensic Science, University of Pavia, 27100 Pavia, Italy; vittoria.carnevalepellino@unipv.it; 2 Department of Industrial Engineering, University of Rome Tor Vergata, 00133 Rome, Italy

Childhood obesity remains a serious public health concern all over the world [1]. Even if obesity has complex and multi-factorial causes, the higher food intake and reduced physical activity (PA) practice are the predominant causes for childhood obesity. Several studies indicated that children with obesity tend to be adults with obesity, with higher risk of developing serious cardiovascular and metabolic diseases and co-morbidity, and consequently, worse quality of life [2,3,4], but timely surveillance and targeted intervention are essential to reduce problematic habits from a young age, with positive effects in adulthood [5,6].

The World Health Organization (WHO) recommends specific PA guidelines for children aged 5–17 years, highlighting the importance of reaching at least 60 min per day of moderate to vigorous PA practice, in order to reach the positive health-related outcomes [7]. These activities include play, games, sports, active transportation, physical education at school and planned sport activities. Despite clear benefits demonstrated from regular PA practice [8,9], children with obesity tend to have lower levels of PA practice and physical fitness [10,11] compared to peers, caused by greater difficulties in performing different motor skills and negative feelings and self-esteem related to PA practice [12]. For these reasons, pediatric and sport specialists should carefully evaluate the barriers to exercise practice and implement PA and sports strategies to augment long-term PA adherence.

Traditional interventions, including periods of aerobic activity at a predetermined intensity, are effective to reduce fat mass and ameliorate cardiovascular profiles [13,14], but overweight and obese young people are less willing, and sometimes even unable, to participate in prolonged periods of endurance training. In fact, children with obesity tend to negatively perceive PA and may find sedentary activities more attractive than young people of normal weight [15]. In addition, excess body fat hinders the performance of natural-load physical activity, such as light running, and can increase the risk of musculoskeletal injuries [16]. These observations underline the importance of considering the type, intensity, frequency and progression of training, when planning interventions for children overweight and with obesity. New information on planning child weight management programs suggests that strength training or combined strength and aerobic training (e.g., circuit or interval training) can offer all young people a safe, effective and valuable method for physical conditioning, regardless of body size [17,18,19]. Previous studies confirmed that participation in complex interventions that are engaging, challenging and fun offer overweight and obese young people the opportunity to feel comfortable with their performance and experience the benefits of physical training [17,20,21]. In fact, children with obesity tend to be relatively strong, as the excess body mass sustained during daily activities appears to act as a chronic training stimulus. As a result, the high rates of adherence to interventions that include resistance training are not surprising, as this type of exercise offers youth with obesity the chance to learn new exercises, to excel and receive unsolicited positive feedback.

Muscle strength has been shown to be inversely related to metabolic risk in young people [22,23] and longitudinal findings indicate that muscle fitness phenotypes (i.e., muscle strength, power and endurance) in childhood can be used to predict metabolic syndrome in adulthood [24]. Since the benefits of strength training for health and fitness markers in children and adolescents have been shown to be independent of cardiorespiratory fitness [25], resistance exercise can be particularly beneficial to start a PA practice [26] and lay the foundations for more advanced interventions when health and training level improve.

Collectively, interventions that combine aerobic and resistance training have been shown to produce favorable changes in fat mass, metabolic profiles and inflammatory status in overweight and obese youth, and these changes tend to be accentuated in higher-intensity programs, which also promote better adherence to exercise [27,28,29]. In addition to improving body composition, as a long-term goal, it is important that healthy behaviors are established in the short term, for example, through opportunities to participate in school and social physical activities, including structured physical training [30].

Therefore, PA programs that include higher-intensity sessions of both aerobic training and strength training may be a useful approach because they are characterized by short periods of PA, interspersed with short rest periods [31,32]. Young people may find high-intensity interval training more enjoyable than continuous aerobic exercise because of the feelings of gratification, activation and success [33]. Finally, sport specialists and researchers have to carefully consider factors that influence obese children to begin and continue an exercise program, including the opportunity to improve strength, fitness and enjoyable social interaction experiences with peers [34], to encourage PA as a lifestyle.
